# Effectiveness of minimally invasive surgery using incomplete phalangeal osteotomy for symptomatic curly toe of adults with a trapezoidal phalanx: An observational study

**DOI:** 10.3389/fsurg.2022.965238

**Published:** 2022-09-20

**Authors:** Leonor Ramírez-Andrés, Eduardo Nieto-García, Elena Nieto-González, Noemí López-Ejeda, Javier Ferrer-Torregrosa

**Affiliations:** ^1^ Doctorate School, Valencia Catholic University “San Vicente Mártir,” Valencia, Spain; ^2^ Podiatry Department, Faculty of Medicine and Health Sciences, Valencia Catholic University “San Vicente Mártir,” Valencia, Spain; ^3^ Unit of Physical Anthropology, Department of Biodiversity, Ecology and Evolution, Faculty of Biological Sciences, Complutense University of Madrid, Madrid, Spain

**Keywords:** curly toe, varus toe, trapezoidal phalanx, dismetric phalanx, minimal incision surgery, incomplete phalangeal osteotomy (IPO), unicortical osteotomy

## Abstract

**Background and aims:**

Digital deformity in flexion, varismus (external rotation), and adduction with the toe in both supraduction and infraduction are called clinocampodactyly or curly toe. All adult patients with symptoms and a diagnosis of semirigid/rigid curly toes underwent radiological examination to verify the presence of a trapezoidal phalanx. The purpose of this study was to quantitatively determine the degrees of improvement of a dysmetric phalanx after incomplete phalangeal osteotomy using minimally invasive surgery. The points of improvement were determined using the American Orthopedic Foot and Ankle Society (AOFAS) scale score.

**Methods:**

Between May 2021 and June 2022, 30 patients diagnosed with curly toes underwent unicortical osteotomy of the affected phalanx. The convergence angle was measured and the AOFAS scale scores were compared.

**Results:**

A total of 33 toes underwent surgery. The average reduction of the convergence angle was 9°. The average improvement in the AOFAS scale score was 53 points at 6 months and reached almost 90 points (89.9 ± 6.1 points).

**Conclusions:**

Incomplete phalangeal osteotomy performed with minimally invasive surgery of the trapezoidal phalanges of curly toes of adults can improve alignment and AOFAS scale scores.

## Introduction

Clinocampodactyly or curly toe is a deformity of the toes in the positions of flexion, varismus (external rotation), and adduction. Patients are able (1–4) to maintain the metatarsophalangeal joint flexed or neutral (5, 6) and place it in supraduction or infraduction of the adjacent joint (7). The triplanar involvement of the deformity is implicit (8).

Curly toe is more frequent in the fourth and fifth toes (1, 3, 9–11) and often appears bilaterally (11, 12). Most cases occur in childhood and are determined by a familial history and/or a congenital deformity (3, 7, 9, 10, 12); however, others may occur in adults due to shoe wearing or an unknown origin. Curly toe is simply diagnosed by clinical exploration and identification of flexible deformities in children (1, 3, 8, 10), while semirigid or rigid deformities are identified in adults (2, 7), although this method can be complemented with x-ray methods or echography (3, 11). Symptoms are diverse, including hyperkeratosis, helomas, onychodystrophies, and limitations in placing shoes normally on the feet. In adults, symptomatic cases are treated (1, 3, 7, 13).

All adult patients diagnosed with semirigid/rigid curly toes using the Kelikian push-up test (14) underwent radiological examination to verify the presence of a trapezoidal phalanx (4, 15, 16). The absence of parallelism between the proximal and distal interphalangeal joints determines the angle proportional to the severity of the curly toe deformity (2, 7). During this study, the degrees of the convergence angle of the proximal and distal articular surfaces were assessed preoperatively for curly toe toes diagnosed with trapezoidal phalanx and postoperatively after incomplete phalangeal osteotomy (IPO) involving minimally invasive surgery (17). They were evaluated using the foot and ankle function scale designed by the American Orthopedic Foot and Ankle Society (AOFAS) (18) before surgery and after 6 months postoperatively.

The objective of this study was to quantitatively determine the degree of improvement in the trapezoidal phalanx after IPO with minimally invasive surgery and AOFAS scale scores.

## Material and methods

This observational and longitudinal study was performed at the E. Nieto Podiatric Clinic in Logroño by several professionals following the same protocol of diagnosis, surgery, and subsequent monitoring. The inclusion criteria were adult patients with radiologically diagnosed symptomatic rigid or semirigid curly toes with trapezoidal phalanges. The exclusion criteria were aged younger than 16 years, radiological images of open physes, previous digital surgery, previous digital fractures, neuromuscular pathology, disfiguring arthritic/arthritic degenerative processes, and asymptomatic or flexible curly toe deformities.

### Participants

The sample comprised 23 women (76.7%) and 7 men (23.3%). The average age was 56.5 ± 15.4 years. The intervention was performed between May 2021 and June 2022. No significant differences were found between genders (women: 55.5 ± 15.7 years; men: 60.1 ± 15.3 years; *p* = 0.338). Regarding clinical history, 16.7% (*n* = 5) had a family history of a similar pathology and 16.7% (*n* = 5) had been diagnosed with diabetes mellitus.

The study was authorized by the Research Ethics Committee of the Universidad Católica de Valencia San Vicente Mártir (registry number UCV/2020-2021/081).

### Method of measuring the angle of convergence of the articular surfaces

**Figure 1:**
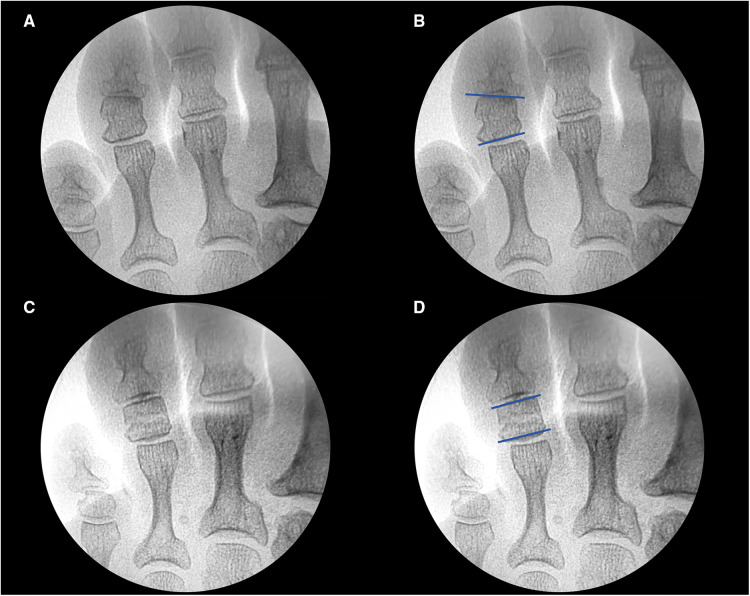
(**A**) Preoperative fluoroscopy. (**B**) Preoperative fluoroscopic measurement of the angulation between the proximal and distal articular surfaces at the level of the middle phalanx of the fourth toe of the left foot. (**C**) Postoperative fluoroscopy after performing IPO. (**D**) Postoperative fluoroscopic measurement of the angulation between the proximal and distal articular surfaces at the level of the middle phalanx of the fourth toe of the left foot.

The angle of convergence of the articular surfaces was determined by the interphalangeal line of the proximal articular surface and the interphalangeal line of the distal articular surface of the trapezoidal phalanx. The greater the angle, the greater the severity of the deformity (Figure 1).

Presurgical angular measurement was performed after minimally invasive techniques with IPO of the phalanx with dysmetria. The AOFAS (18) questionnaire was completed on the preoperative day and 6 months postoperatively. The results were considered to be excellent (90–100 points), good (80–89 points), medium (70–79 points), or poor (less than 70 points).

Of the 30 patients, 2 presented with bilateral deformity and 2 presented with deformity in three toes of the same foot. Of the 36 osteotomies performed, 19 involved the fourth toe (7 right and 12 left toes), 11 involved the third toe (5 right and 6 left toes), and 6 involved the second toe (2 right and 4 left toes).

### Surgical technique

To perform a unicortical wedge osteotomy with minimal incision surgery, 2% mepivacaine was used for local anesthesia, along with small skin incisions, and no ankle tourniquet or fixation. The use of specific instrumentation, such as a fluoroscope to control bone position and a low-speed high torque drill (4:1) with a handpiece to avoid the risk of osteonecrosis and burns, is necessary for the successful execution of this technique (19–23).

**Figure 2:**
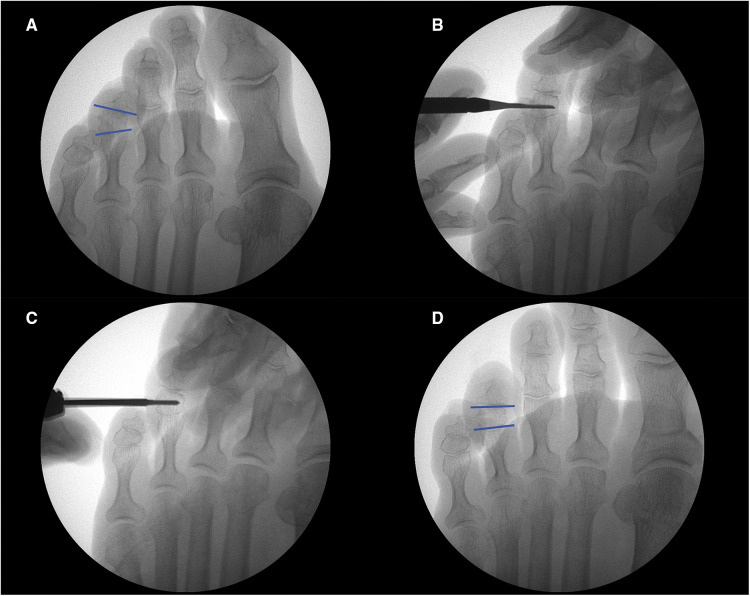
(**A**) preoperative fluoroscopy. (**B**) Skin incision with 64 MIS scalpel blade. (**C**) Performing an incomplete osteotomy over the middle phalanx with a Shannon 2.0 mm × 8.0 mm burr. (**D**) Postoperative result.

**Figure 3:**
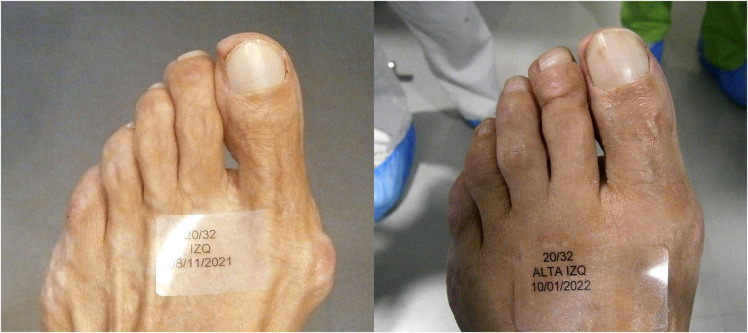
Dorsal pre and postoperative images of the left fourth toe.

**Figure 4:**
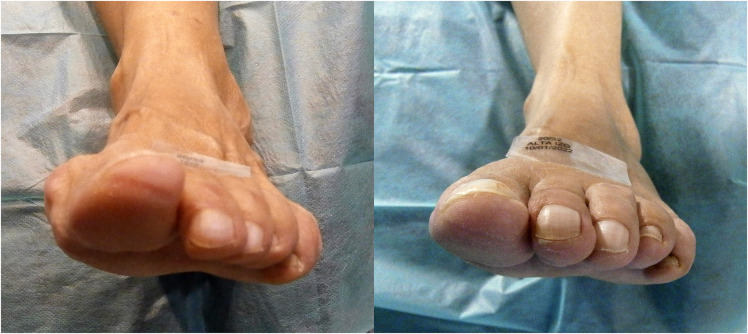
Frontal pre and postoperative images of the left fourth toe. Note the changing position of the nail.

The skin was incised dorsolaterally to the trapezoidal phalanx under fluoroscopic control with a Beaver 64 MIS scalpel blade while avoiding risk of injury to the vasculonervous bundle. The Shannon 2.0 × 8.0 mm burr was used to perform unicortical osteotomy using the quadrant theory (13, 17, 19, 24, 25). The wedge opening is made opposite to the toe deformity while keeping a part of the cortex intact, to allow performing the closing osteotomy and avoid displacement. Its spatial orientation is individually designed for each case depending on previous toe misalignment; however, maintaining the hinge intact is mandatory (Figures 2–4).

**Figure 5:**
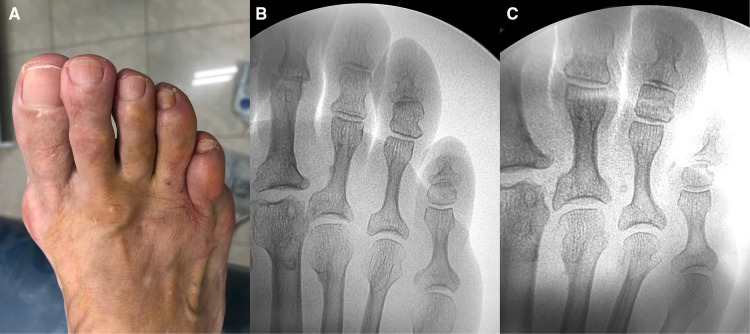
(**A**) and (**B**) Preoperative photograph and fluoroscopy with trapezoidal middle phalanx at the level of the right fourth toe. (**C**) Postoperative fluoroscopy of the unicortical digital osteotomy over the phalanx. Note the peroneal and tibial cortical balance.

Radiological imaging showed that the phalangeal cortices with dysmetria before surgery no longer exhibited dysmetria, indicating the achievement of realignment of the digit in the three planes (Figure 5). If intraoperative realignment was not observed, then soft tissue involvement in the deformity was indicated (24). For these cases, tenotomy of the flexor digitorum longus, flexor digitorum brevis, and/or the extensor digitorum longus following the same protocol for digital deformities is necessary (7, 17).

Under x-ray control, the finger was splinted postoperatively using a nonwoven adhesive bandage to maintain correct alignment and closure of the osteotomy of the phalanx that underwent surgery. The bandage was maintained during the primary consolidation period. Weekly radiological evaluations were performed. The bandage was removed after 3–4 weeks. Patients were able to normally place shoes on their feet 45 days postoperatively. Regarding physical activity, weight-bearing is possible during the first 48 h but with adequate resting time. Activity must be gradually increased during this period.

### Sample size

The sample size was calculated using GRANMO version 7.12 online software developed by the Institut Municipal d'Investigació Mèdica de Barcelona. The formula established for the comparison of paired means (repeated in the same group) was considered, introducing an alpha risk of 5% and statistical power of 90% (beta risk of 10%). The unilateral hypothesis of reduction in the alignment angle of the intervened phalanges was expected to be verified. We used reference values reported by Choi et al. (2), who applied similar methodology and found a mean reduction of 31° ± 16° after a minimally invasive intervention. According to these parameters, the minimum required sample size was three patients. However, to facilitate the statistical analysis, the sample size was expanded to a minimum of 30 patients.

### Statistical analysis

Statistical analysis was performed using SPSS version 27. The distribution of continuous variables was verified using the Kolmogorov–Smirnov test with Lilliefors correction. To compare variables with normal distributions between two independent groups, Student's *t*-test was performed; for non-normal variables, the Mann–Whitney *U* test was performed. When there were more than three groups, such as for the typology of the foot or intervened toe, an analysis of variance (ANOVA) or Kruskal–Wallis test was performed, respectively. To compare preoperative and postoperative results, paired analysis tests, such as the Wilcoxon test for non-normal variables and paired Student's *t*-test for normal variables, were performed. Simple linear regression analyses were performed to verify the association between continuous variables. During all analyses, *p* < 0.05 was considered significant.

## Results

Of the 30 participants in this study, 26 (86.7%) underwent surgery on a single toe, 2 (6.7%) underwent bilateral intervention on one toe of each foot, and 2 (6.7%) underwent intervention on three toes of the same foot (left foot for both patients). After the push-up test examination, all symptoms were diagnosed as semirigid and rigid curly toe deformities.

Of the 36 osteotomies, 61.1% (*n* = 22) involved Greek typology, 30.6% (*n* = 11) involved square typology, and 8.3% (*n* = 3) involved Egyptian typology. Regarding laterality, 61.1% (*n* = 22) were on the left foot, with L4 being the most frequent (*n* = 12; 54.5%), followed by L3 (*n* = 6; 27.3%) and L2 (*n* = 4; 18.2%). Of the 14 interventions of the right foot (38.9%), half (*N* = 7; 50.0%) were performed on R4, 35.7% (*n* = 5) were performed on R3, and 14.3% (*n* = 2) were performed on R2.

Regarding the behavior of the deformity when performing the push-up test, 63.9% (*n* = 23) were irreducible and 36.1% (*n* = 13) showed a partial reduction. Finally, 72.2% (*n* = 26) exclusively underwent incomplete osteotomy and 27.8% (*n* = 10) underwent osteotomy associated with flexor or extensor tenotomy.

Table 1 shows the comparison of preoperative and postoperative results of the angle of convergence of the intervened toes and scores of three components of the AOFAS scale (pain, functionality, and alignment). All variables included in the study showed an average reduction of 9° in the angulation and an average increase of 53 points in the AOFAS scale score 6 months after the intervention; furthermore, this score reached close to 90 out of 100 after the intervention. However, when analyzing the association between both variables using a simple linear regression analysis, no significant associations between the variation in the AOFAS scale score (dependent variable) and the variation in angulation (independent variable) (*p* = 0.929) were observed.

**Table 1:** Comparison of results before and after surgery.

	Before surgery	After surgery	Differences	*p*-value
	Mean range (SD)	Mean range (SD)	Mean range (SD)	
Angulation (degrees)	12.3 (5.8)	3.2 (3.5)	−9.1 (6.7)	<0.001
AOFAS scale				
Total score	37.0 (16.5)	89.9 (6.1)	+52.9 (17.4)	<0.001
Pain	9.4 (10.1)	36.7 (4.8)	+27.2 (11.9)	<0.001
Functionality	21.6 (7.5)	39.0 (4.5)	+17.4 (8.9)	<0.001
Alignment	5.1 (4.6)	13.3 (3.1)	+8.2 (4.0)	<0.001

AOFAS, American Orthopedic Foot and Ankle Society; SD, standard deviation.

Table 2 shows the comparison of differences in the angulation and AOFAS scale score after the intervention based on the characteristics of the patient and the injury. The difference in angulation was only significantly associated with the result of the push-up test; therefore, partially reducible lesions showed a much greater change in angulation compared to irreducible lesions (−13° vs. −7°). The differences in the AOFAS scale scores were associated with the patient's gender, with greater improvement observed for women (+54 vs. +40 points for men), and the Kelikian push-up test results; partially reducible lesions showed greater improvement (+62 vs. +45 points for irreducible lesions). No significant associations were observed for any of the other variables.

**Table 2:** Comparison of postoperative results according to patient profiles and characteristics of the injury and surgery.

Analyzed variables		Angulation difference	AOFAS difference
		Mean range (SD)	Mean range (SD)
Sex	Female	−10.0 (7.3)	+56.7 (17.8)
	Male	−6.8 (4.4)	+43.2 (12.3)
	*p*-value	0.127	0.017*
Age	≤60 years	−7.6 (5.9)	+55.9 (16.6)
	>60 years	−10.1 (7.4)	+51.1 (18.2)
	*p*-value	0.259	0.525
Diabetes mellitus	Yes	−8.6 (4.5)	+55.4 (22.9)
	No	−9.2 (7.1)	+52.5 (16.8)
	*p*-value	0.818	0.894
Foot typology	Greek	−9.6 (6.8)	+55.6 (15.8)
	Egyptian	−15.6 (0.8)	+40.3 (6.8)
	Square	−6.2 (6.2)	+51.0 (21.4)
	*p*-value	0.081	0.226
Foot laterality	Right	−8.3 (6.5)	+53.6 (20.1)
	Left	−9.4 (7.0)	+54.4 (15.4)
	*p*-value	0.613	0.611
Operated toe	Toe 2	−6.5 (7.4)	+57.5 (7.0)
	Toe 3	−5.9 (4.3)	+53.2 (22.5)
	Toe 4	−11.7 (6.9)	+53.5 (16.5)
	*p*-value	0.051	0.713
Push-up test results	Irreductible	−7.1 (5.7)	+47.1 (14.9)
	Partial reduction	−12.7 (7.1)	+63.3 (17.0)
	*p*-value	0.014*	0.004**
Intervention	Incomplete osteotomy	−8.6 (6.8)	+53.1 (18.2)
	Osteotomy plus tenotomy	−10.4 (6.8)	+52.5 (16.0)
	*p*-value	0.448	0.958

Age^a^ divided by the sample mean value (60 years).

* Significance considered when *p* < 0.05.

** Significance considered when *p* < 0.01.

AOFAS, American Orthopedic Foot and Ankle Society; SD, standard deviation.

There were no postoperative complications of infection. Reintervention was necessary for only one patient with pain and temporary postoperative edema. Postoperative stiffness was not a direct consequence of the intervention performed for the phalanx with dysmetria.

## Discussion

Curly toe is a three-dimensional digital deviation of flexion, varus, and adduction. It can be diagnosed by x-ray examination, confirming a trapezoidal phalanx with a dysmetric peroneal and tibial cortex, with no parallelism between the proximal and distal interphalangeal joints. In adults, acquired deformities can be evaluated (2, 6).

Hamer's visual classification (3, 11, 26) or appearance criteria (good, fair, or bad) (27, 28) are used to determine surgical procedures during open-field surgery. In 2021, Satake et al. proposed an objective classification that correlated dorsoplantar radiograph results and physical examination results (26). To determine the severity criteria, Lee et al. (4) performed angular measurements of the axes of the proximal phalanx and distal phalanx. During our study, we determined the classification using the angulation of convergence of the articular surfaces and considered resolution when it was close to 0°.

Regarding treatment, using syndactyly-type bandages is considered effective for children when the deformity is flexible (28–31). Surgery is reserved for symptomatic cases, nonflexible cases, and cases that may cause future complications (1, 26, 27, 29).

Soft tissue surgery with flexor to extensor transfer (8, 32) or tenotomy (involving the flexor digitorum longus and brevis) in young people with deformities (1, 5, 9, 27) has shown good results. Hamer and Standley (10) found no statistical differences between flexor to extensor transfer and tenotomy in a double-blind and randomized study. Jacobs and Vandeputte got better results using a z lengthening of the flexor digitorum longus adding a tenotomy of the flexor digitorum brevis if it is necessary (3).

Bone surgery has been defended by numerous authors. Zafiropoulos and Henry found better results from bone straightening and less weakness with a wedge osteotomy, as did Kirschner wire (7, 15). In 2015, Choi et al. (2) performed a dorsolateral closing wedge-shaped resection arthroplasty, and in 2021, Lee et al. (4) expressed a preference for minimally invasive techniques because of their better postoperative results.

Various authors have incorporated minimally invasive techniques with refrigeration, tourniquet, osteosynthesis, and rigid splinting (4, 24, 33–35). During our study, we only performed unicortical osteotomy and bandaging, which showed good results (23).

Due to the involvement of bony parts in bone deformities, soft tissue techniques are not effective. IPO with minimally invasive techniques may be altered for each patient, to achieve a balance of the phalangeal cortex with the realignment of the toes. There is no length or distal vs. proximal deformity references postulated by Choi et al. (2). Bone techniques using IPO were prioritized, and if no reducibility was observed, then tenotomy was performed (17). The effectiveness of the technique described was the same, even if there was concomitant joint involvement.

Zafiropoulos and Henry (15) determined a value of 10° postoperatively as the limit when assessing varus persistence in curly toes. Choi recommended valgus surgical overcorrection of 10° to prevent recurrence (4). In contrast, during this study, after performing IPO with minimally invasive surgery, a significant improvement was obtained with an average angulation correction of 9°.

Only three articles referred to the use of the AOFAS scale to control improvement: Nieto-García et al. (17) who showed 95.7 points of recovery at the 12-month follow-up, and Yassin (34) who demonstrated 70 points at the 6-month follow-up. Jacobs and Vandeputte (3) refer to an improvement in the AOFAS scale but do not specify the points of recovery. The results of our study showed an average of 89.9 points at the 6-month follow-up.

We consider IPO with minimal incision surgery to be a satisfactory technique that achieves good realignment of the toe with the trapezoidal phalanx, preserving the adjacent joints for improved patient comfort postoperatively, having a low rate of complications and a high level of patient satisfaction.

### Study limitations

During this descriptive and observational study, objective numerical values of the convergence angle were determined without establishing severity criteria based on the angulation, which could serve as a basis for future research. Another limitation was the short monitoring time of 6 months.

## Conclusions

Minimally invasive surgery is the technique of choice for symptomatic semirigid or rigid curly toes of adults with trapezoidal phalanx involvement because it preserves the joint, results in a high level of postoperative satisfaction, improves functionality with little postoperative pain, and is associated with few complications.

## Data Availability

The raw data supporting the conclusions of this article will be made available by the authors, without undue reservation.
